# Epidemiological trends, prognostic factors, and survival outcomes of synchronous brain metastases from 2015 to 2019: a population-based study

**DOI:** 10.1093/noajnl/vdad015

**Published:** 2023-03-05

**Authors:** Megan Parker, Kelly Jiang, Jordina Rincon-Torroella, Joshua Materi, Tej D Azad, David O Kamson, Lawrence R Kleinberg, Chetan Bettegowda

**Affiliations:** Department of Neurosurgery, Johns Hopkins University School of Medicine, Baltimore, Maryland, USA; Department of Neurosurgery, Johns Hopkins University School of Medicine, Baltimore, Maryland, USA; Department of Neurosurgery, Johns Hopkins University School of Medicine, Baltimore, Maryland, USA; Department of Neurosurgery, Johns Hopkins University School of Medicine, Baltimore, Maryland, USA; Department of Neurosurgery, Johns Hopkins University School of Medicine, Baltimore, Maryland, USA; Department of Neurology, Brain Cancer Program, Johns Hopkins University School of Medicine, Baltimore, Maryland, USA; Department of Radiation Oncology and Molecular Radiation Sciences, Johns Hopkins University, Baltimore, Maryland, USA; Department of Neurosurgery, Johns Hopkins University School of Medicine, Baltimore, Maryland, USA

**Keywords:** brain metastases, incidence, prognosis, SEER, survival

## Abstract

**Background:**

Brain metastases (BM) constitute a significant cause of oncological mortality. Statistics on the incidence of BM are limited because of the lack of systematic nationwide reporting. We report the incidence of synchronous brain metastases (sBM), defined as BM identified at the time of primary cancer diagnosis from 2015 to 2019 using National Cancer Institute's (NCI’s) Surveillance, Epidemiology, and End Results Program database.

**Methods:**

We identified 1,872,057 patients with malignancies diagnosed between 2015 and 2019 from the SEER 17 Registries database, including 35,986 (1.9%) patients with sBM. Age-adjusted incidence rates were examined using the NCI Joinpoint software. Kaplan-Meier curves and a multivariate Cox regression model were used to investigate survival.

**Results:**

The incidence rate of sBM from 2015 to 2019 was 7.1 persons per 100,000. Lung and bronchus cancers had the highest incidence of sBM (5.18 to 5.64 per 100,000), followed by melanoma (0.30 to 0.34 per 100,000) and breast cancers (0.24 to 0.30 per 100,000). In children, renal tumors had the highest sBM incidence. sBM were associated with poorer survival than extracranial metastases only (hazard ratio [HR]: 1.40 [95% CI: 1.39–1.42], *P* < .001). We observed better survival in white patients relative to nonwhite patients with sBM (HR: 0.91 [95% CI: 0.90–0.94], *P* < .001).

**Conclusions:**

The incidence rate of sBM has remained similar to rates reported over the last 9 years, with the majority associated with primary lung and bronchus cancers. sBM represent a national healthcare burden with tremendous mortality in pediatric and adult populations. This population may benefit from improved screening and treatment strategies.

**Key Points**

- The incidence of synchronous brain metastases (sBM) from 2015 to 2019 was 7.1 persons per 100,000.
- Patients with sBM have lower survival than patients with extracranial metastases only.
- Sociodemographic characteristics impact survival in patients with sBM.

**Importance of the Study**

Utilizing the most recently released SEER registry, we report the incidence, demographics, and prognosis of patients with brain metastases identified at the time of primary cancer diagnosis in the United States from 2015 to 2019. Our study builds on two SEER-based epidemiologic studies of synchronous brain metastases in the United States published between 2010 and 2015. We noted a stable incidence of synchronous brain metastases of approximately 7 persons per 100,000 over the 5-year study period. We also found significant demographic and clinical prognostic factors among patients with metastatic disease. The results of this study may help inform physicians, patients, and public health systems when confronting malignant disease and brain metastases.

Brain metastases (BM) occur in 10%–20% of adults with malignancies, and they are 10 times more common than primary brain cancers.^1^ Despite the large burden of morbidity and mortality, the epidemiological characterization of BM is limited because, unlike other cancers, there is no systematic nationwide reporting of secondary brain malignancies. Most estimates come from an amalgam of sources such as autopsy reports, death certificates, cancer registries, and institutional records which have smaller sample sizes and may lack accuracy due to regional and sampling biases. Several population-based studies have suggested that the incidence of BM has risen over the last several decades and is now estimated to be 8.3 to 14.3 per 100,000 population.^2,3^ The prevalence of BM reported in autopsy studies ranges from 9% to 26% of patients with cancer.^4,5^ However, these studies are decades old and precede current advanced imaging techniques and treatment options, which have improved disease detection and prolonged the lifespan of patients with metastatic disease. As BM awareness rises, it is increasingly important to accurately characterize epidemiologic trends. These studies are necessary to inform patient care via modification to surveillance and diagnostic strategies, resource allocation, and the initiation of preventive measures. Moreover, the results of these studies provide insight into the impact of BM on patients’ cancer progression and overall survival (OS).

In the United States, there are currently no nationwide population-based studies that accurately depict the burden of BM. Estimates have been obtained by identifying synchronous rates, defined by a diagnosis of BM at the time of primary cancer diagnosis. The presence of synchronous brain metastases (sBM) as well as synchronous metastases at other sites is reported in the National Cancer Institute (NCI) Surveillance, Epidemiology, and End Results (SEER) Program, a mandated reporting cancer registry that includes all primary cancers.^6^ In 2020, Singh et al. published an epidemiological analysis on sBM reported in SEER during 2010–2015.^7^ They report the incidence of BM at 7 per 100,000, with 80% originating from primary lung cancer. Here, we conducted an analysis of the most current SEER database with the primary aims to report the incidence of sBM by primary site from 2015 to 2019, to compare survival outcomes of patients with sBM to patients with synchronous extracranial metastases alone, and to investigate the demographics of patients with sBM.

## Materials and Methods

### Data Collection

The SEER 17 registry provides data from 17 population-based registries, representing approximately 26.5% of the US population.^8^ Clinical and demographic data at the time of diagnosis and survival data were collected for all patients. Deidentified data were made publicly available via the NCI SEER program. This study was completed according to the Strengthening the Reporting of Observational Studies in Epidemiology reporting guidelines and approved by the Johns Hopkins Hospital institutional review board (IRB00324993).^9^

We identified 2,164,978 patients diagnosed with primary malignant cancer between January 1, 2015 and December 31, 2019 from the SEER 17 Registries database. Patients with benign/noninvasive or in situ tumors were not included. Patients were excluded if sBM status was unknown (*N* = 292,266, 13.5%). Data on patients’ follow-up and survival time were required for our survival analyses. NCI excludes patients with 0 days of survival from the SEER database. We excluded an additional 655 patients diagnosed by autopsy or death certificate alone as well as patients with unknown follow-up (*N* = 655, 0.03%), leaving 1,872,057 patients (86.5% of the initial cohort) for analysis.

### Case Definitions

SEER registries only report information on synchronous metastases. We characterized patients by the presence or absence of BM at diagnosis of primary cancer, as defined by the *SEER Combined Mets at DX-brain* variable. Patients were further categorized as follows: patients with malignant disease without metastases, those with sBM only, those with sBM and synchronous extracranial metastases at one or more distant sites (lung, liver, bone, distant lymph nodes, and/or other), and those with only synchronous extracranial metastases at one or more distant sites.^7,10^

### Statistical Analyses

We used the NCI SEER*Stat version 8.4.0 to calculate incidence rates age-adjusted to the 2000 US standard population and reported per 100,000 persons, based on the availability of US Census data in the SEER*Stat software and previous SEER-based studies.^7,10^ The Joinpoint Trend Analysis Software, version 4.9.1.0 was used to calculate average annual percent changes (AAPC) in incidence rates.

Demographics were categorized by age at diagnosis, race, sex, and median household income. Based on The Central Brain Tumor Registry of the United States guidelines, age at diagnosis was categorized as children (0–14 years), adolescents and young adults (15–39 years), and older adults (40+ years).^11^ Patients were also stratified by cancer type, according to the *SEER ICD-O-3 Site/Type* variable, which characterizes primary tumor sites according to the World Health Organization’s *International Histological Classification of Tumor* 5th edition, published in 2021.^12^ Patients with primary lung and bronchus tumors were further categorized as small-cell or non-small cell according to the SEER *Histologic Type ICD-O-3* variable. Patients with primary breast cancer were sub-stratified by hormone-receptor (HR) status and human epidermal growth factor receptor 2 (HER2) status.

Additional analyses were performed using the IBM SPSS Statistics software, version 28 (SPSS Inc., Chicago, IL, USA). Numerical, categorical, and time-to-event variables were compared with Wilcoxon Rank Sum, chi-squared tests, and log-rank tests, respectively. One-way ANOVA was used to compare means between the 3 groups. For the survival analysis, the primary exposure was the presence of sBM alone, sBM with synchronous extracranial metastases, or synchronous extracranial metastases alone as a categorical variable, and the main outcome was OS. Median survival time was estimated using the Kaplan-Meier method. A multivariate Cox proportional hazards regression was used to compare the hazards of death for patients with sBM versus patients with extracranial metastases only. Age at diagnosis (continuous), sex (dichotomous), race (categorical), and year of diagnosis (categorical) were included in the multivariate regression as covariates. The Bonferroni correction for multiple comparisons was applied to comparisons of patients with sBM to patients with extracranial metastases alone. We tested 7 hypotheses, yielding a limit of 0.007 for statistical significance. Similar to Singh et al., we did not apply the Bonferroni correction to analyses stratified by primary site, which had a limit of statistical significance of *P* = .05.^7^ Statistical tests were 2-sided.

## Results

### Population Characteristics Stratified by the Presence of SBM, Synchronous Extracranial Metastases Alone, or No Synchronous Metastases

A total of 1,872,057 patients diagnosed with malignant cancer from 2015 to 2019 were included in the analysis, of whom 35,986 (1.9%) had sBM, 259,001 (13.8%) had extracranial metastases alone, and 1,577,070 (84.2%) did not have any metastases at diagnoses. Median age of the entire cohort was 65 years (interquartile range [IQR]: 55–70), with over 94% of patients being 40 years or older. The study population included 947,727 (50.6%) female, 1,278,947 (68.3%) White non-Hispanic, 188,526 (10.1%) Black non-Hispanic, 225,956 (12.1%) Hispanic, 141,939 (7.6%) Asian/Pacific Islander, and 11,557 (0.6%) American Indian/Alaska Native patients. The median follow-up time was 21 months (IQR: 7–38).

A summary of patient characteristics stratified by the presence of sBM, extracranial metastases alone, and no synchronous metastases can be found in Table 1. Compared to patients with extracranial metastases only, patients with BM were younger (63.7 ± 11.8 years vs 65.2 ± 13.6 years, *P* < .001), had lower rates of liver metastases (22.6% vs 39.5%, *P* < .001) and distant lymph node metastases (14.6% vs 17.5%, *P* < .001), and higher rates of bone metastases (36.4% vs 33.7%, *P* < .001). The frequency of lung metastases was roughly 29% in both patients with BM and patients with extracranial metastases alone. We also observed demographic differences in patients with BM compared to patients with extracranial metastases alone. Patients with sBM were more likely to be female (48.1% vs 46.4%, *P* < .001).

**Table 1.** Characteristics of Patients with SBM, Extracranial Metastases Alone, or No Metastases at Diagnosis

	**Brain Metastases** **(*N* = 35,986)**	**Extracranial Metastases Alone (*N* = 259,001)**	**No Metastases** **(*N* = 1,577,070)**	***P***
Age, Mean ± SD	63.7 ± 11.8	65.2 ± 13.6	62.0 ± 14.3	**<.001**
Age category				
Children	99 (0.3%)	958 (0.4%)	6656 (0.4%)	**<.001**
Adolescents and young adults	724 (2.0%)	8217 (3.2%)	86,050 (5.5%)	**<.001**
Older adults	35,163 (97.7%)	24,9826 (96.5%)	1,484,364 (94.1%)	**<.001**
Sex				**<.001**
Females	17,292 (48.1%)	120,079 (46.4%)	810,356 (51.4%)	
Males	18,694 (51.9%)	138,922 (53.6%)	766,714 (48.6%)	
Race				
White	28,363 (78.8%)	202,631 (78.2%)	1,259,158 (79.8%)	**<.001**
Black	4027 (11.2%)	31,602 (12.2%)	156,166 (9.9%)	**<.001**
Asian or Pacific Islander	3197 (8.9%)	21,446 (8.3%)	119,430 (7.6%)	**<.001**
American Indian/Alaska Native	271 (0.8%)	2017 (0.8%)	10,324 (0.7%)	**<.001**
Year of diagnosis				
2015	6895 (19.2%)	38,111 (14.7%)	302,708 (19.2%)	**<.001**
2016	7049 (19.6%)	54,745 (21.1%)	306,882 (19.5%)	**<.001**
2017	7537 (20.9%)	56,481 (21.8%)	314,453 (20.0%)	**<.001**
2018	7184 (20.0%)	54,485 (21.0%)	323,724 (20.5%)	**<.001**
2019	7321 (20.3%)	55,179 (21.3%)	329,303 (20.9%)	**<.001**
Primary site				
Lung and bronchus	27,585 (76.7%)	70,185 (27.1%)	132,747 (8.4%)	**<.001**
Small cell	4369 (12.1%)	11,790 (4.6%)	9528 (0.6%)	**<.001**
Non-small cell	1474 (4.1%)	3144 (1.2%)	4983 (0.3%)	**<.001**
Melanoma	1513 (4.2%)	2551 (1.0%)	107,079 (6.8%)	**<.001**
Breast	1369 (3.8%)	16,156 (6.2%)	306,497 (19.4%)	**<.001**
HR-/HER2-	240 (0.7%)	1817 (0.7%)	29,955 (1.9%)	**<.001**
HR-/HER2+	156 (0.4%)	1137 (0.4%)	11,698 (0.7%)	**<.001**
HR+/HER2-	538 (1.5%)	8881 (3.4%)	218,571 (13.9%)	**<.001**
HR+/HER2+	211 (0.6%)	2265 (0.9%)	30,116 (1.9%)	**<.001**
Kidney and renal pelvis	1100 (3.1%)	9266 (3.6%)	68,663 (4.4%)	**<.001**
Colorectal	524 (1.5%)	33,759 (13.0%)	140,076 (8.9%)	**<.001**
Esophagus	381 (1.1%)	5593 (2.2%)	13,128 (0.8%)	**<.001**
Pancreas	268 (0.7%)	27,676 (10.7%)	30,694 (1.9%)	**<.001**
Prostate	237 (0.7%)	19,914 (7.7%)	231,436 (14.7%)	**<.001**
Stomach	207 (0.6%)	9406 (3.6%)	21,871 (1.4%)	**<.001**
Liver	138 (0.4%)	4651 (1.8%)	30,913 (2.0%)	**<.001**
Urinary bladder	119 (0.3%)	3548 (1.4%)	83,528 (5.3%)	**<.001**
Testis	117 (0.3%)	1220 (0.5%)	10,976 (0.7%)	**<.001**
Thyroid	95 (0.3%)	1447 (0.6%)	61,322 (3.9%)	**<.001**
Cervix	64 (0.2%)	1869 (0.7%)	14,398 (0.9%)	**<.001**
Ovary	74 (0.2%)	5899 (2.3%)	19,314 (1.2%)	**<.001**
Metastases				
Brain	35,986 (100.0%)	0		
Bone	13,091 (36.4%)	87,269 (33.7%)		**<.001**
Lung	10,451 (29.0%)	76,624 (29.6%)		.911
Liver	8123 (22.6%)	102,352 (39.5%)		**<.001**
Distant lymph node	5269 (14.6%)	45,347 (17.5%)		**<.001**
Other	8587 (23.9%)	74,339 (28.7%)		**<.001**

HR, hormone receptor; HER2, human epidermal growth factor receptor 2. Values are expressed as mean ± standard deviation or number (column %), unless indicated otherwise. Bold type indicates statistical significance.

**Figure 1.**
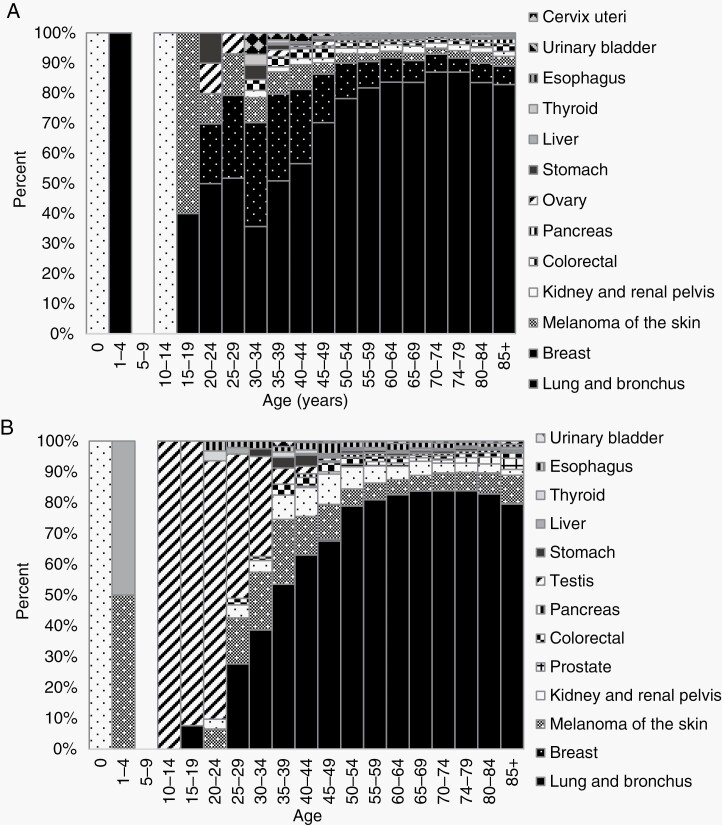
Frequency of sBM by primary site and age in (A) females and (B) males from SEER 2015–2019.

Primary tumors associated with sBM varied by age and sex, as shown in Figure 1. Males with sBM younger than 1 year were more likely to have kidney and renal pelvis tumors, whereas 10–34 year old males with sBM were more likely to have testicular cancer. Females with sBM aged 0–14 years were more likely to have kidney and renal pelvis tumors, whereas melanoma of the skin and breast cancer were more commonly associated with sBM in females 15–39 years old. Lung and bronchus cancers were the most common primary sites associated with sBM for both males and females 35–85+ years. In overall comparison to females with sBM, males with sBM were more likely to have kidney and renal pelvis tumors (4.4% vs 1.9%, *P* < .001), melanoma of the skin (12.4% vs 2.7%, *P* < .001), esophageal cancer (1.8% vs 0.3%, *P* < .001), and stomach cancer (0.7% vs 0.3%, *P* < .001).

Rates of sBM also varied by race and primary tumor site (Table 2). Rates of lung and bronchus cancers with sBM were significantly higher in Asian or Pacific Islander patients than in patients of other races (84.3% vs 75.9%, *P* < .001). Melanoma with sBM were more common among white patients than nonwhite patients (5.2% vs 0.3%, *P* < .001).

**Table 2.** Percent of Patients with SBM by Primary Site and Race

	Race				
Primary Tumor Site	White (%)	Black (%)	American Indian/Alaska Native (%)	Asian or Pacific Islander (%)	*P-*Value
Lung and bronchus	75.3	81.2	66.8	84.3	<.001
Melanoma	5.2	0.3	0.7	0.5	<.001
Breast	3.6	5.0	5.9	3.5	<.001
Kidney and renal pelvis	3.3	2.3	5.5	1.9	.001
Colorectal	1.5	1.6	2.2	1.1	.015
Esophagus	1.2	0.6	3.0	0.5	.024
Pancreas	0.7	0.8	1.1	0.8	.502
Prostate	0.6	1.1	0.7	0.3	.028
Stomach	0.6	0.4	2.2	0.6	<.001
Liver	0.4	0.5	0.7	0.3	.167
Urinary bladder	0.4	0.1	0.7	0.1	.129
Ovary	0.2	0.2	0.4	0.2	.778
Thyroid	0.2	0.3	0.0	0.6	.006
Cervix	0.2	0.3	0.0	0.1	.428

Among patients with sBM, 23,889 (66.4%) also had extracranial metastases at diagnosis, while 8484 (23.6%) had sBM only. The status of extracranial metastases was unknown in 3613 (10.0%) patients with sBM. A comparison of patients with sBM only to patients with sBM and synchronous extracranial metastases can be found in Supplementary Table 1. Patients with sBM only were older than patients who presented with both BM and extracranial metastases at diagnosis and were significantly more likely to be Black or American Indian/Alaska Native. Patients with sBM alone were more likely to have lung and bronchus neoplasms than patients with concurrent BM and extracranial metastases at diagnosis.

### Trends in the Incidence of SBM

The incidence rate of sBM from 2015 to 2019 was 7.1 persons per 100,000 and was relatively stable over the study period. Trends in sBM by the primary tumor site are presented in Table 3. Lung and bronchus cancers consistently had the highest incidence of sBM (ranging from 5.18 to 5.64 per 100,000 or 76.7% of all sBM), followed by melanoma of the skin (0.30 to 0.34 per 100,000) and breast cancers (0.24 to 0.30 per 100,000). Among patients with primary lung and bronchus malignancies, those with small-cell lung cancer had a higher incidence of sBM than those with non-small-cell cancer (0.84 vs 0.29). We observed a significant increase in the incidence of sBM associated with colorectal cancers (AAPC = 5.7%, *P =* .028) from 2015 to 2019.

**Table 3.** Trends in SBM by Primary Site From 2015 to 2019

	Incidence Rates^1^							
Primary Tumor Site	2015–2019	2015	2016	2017	2018	2019	AAPC (%)	*P-*value
All Sites	7.11	7.11	7.10	7.45	6.93	6.95	−0.7	.519
Lung and bronchus	5.40	5.60	5.36	5.64	5.24	5.18	−1.8	.164
Melanoma	0.31	0.30	0.30	0.30	0.31	0.34	3.5	.058
Breast	0.28	0.29	0.24	0.30	0.27	0.29	1.4	.666
Kidney and renal pelvis	0.22	0.21	0.22	0.23	0.21	0.22	−0.3	.804
Colorectal	0.11	0.10	0.10	0.10	0.12	0.12	5.7	.028
Esophagus	0.07	0.08	0.07	0.07	0.06	0.08	−1.1	.799
Pancreas	0.05	0.02	0.07	0.07	0.06	0.05	3.4	.822
Prostate	0.05	0.04	0.05	0.05	0.05	0.05	6.3	.135
Stomach	0.04	0.05	0.04	0.05	0.03	0.04	−6.5	.340
Liver	0.03	0.03	0.03	0.03	0.03	0.02	−2.0	.706
Urinary bladder	0.02	0.03	0.02	0.02	0.02	0.03	3.5	.610
Ovary	0.02	0.02	0.02	0.01	0.01	0.01	−10.7	.194
Thyroid	0.02	0.03	0.03	0.01	0.01	0.02	−4.2	.724
Cervix	0.01	0.02	0.01	0.01	0.02	0.01	−3.5	.663

AAPC, average annual percent change.

^1^All incidence rates are per 100,000 and age-adjusted to the 2000 US standard population.

We also described the incidence of sBM from 2015 to 2019 by age category, depicted in Supplementary Table 2. The incidence of sBM over the study period was 0.14 per 100,000 persons in children, 0.51 per 100,000 persons in adolescents and young adults, and 16.03 per 100,000 persons in older adults. The incidence of sBM was highest in older adults across all primary sites. Lung and bronchus cancers had the highest incidence of sBM in adolescents/young adults and older adults (0.18 and 12.40, respectively). Following lung and bronchus, breast (0.07), melanoma (0.05), renal (0.01), and colorectal (0.01) cancers had the highest incidence of sBM in adolescents and young adults. A similar trend was observed in older adults, with melanoma (0.68), breast (0.60), renal (0.50), and colorectal (0.24) cancers having the highest incidence of sBM after lung and bronchus cancers. In children, kidney and renal pelvis cancers had the highest incidence of sBM (0.005), followed by lung and bronchus (0.002), melanoma (0.001), and liver cancers (0.001).

### Survival of Patients With sBM Only, sBM With Synchronous Extracranial Metastases, and Synchronous Extracranial Metastases Alone

**Figure 2.**
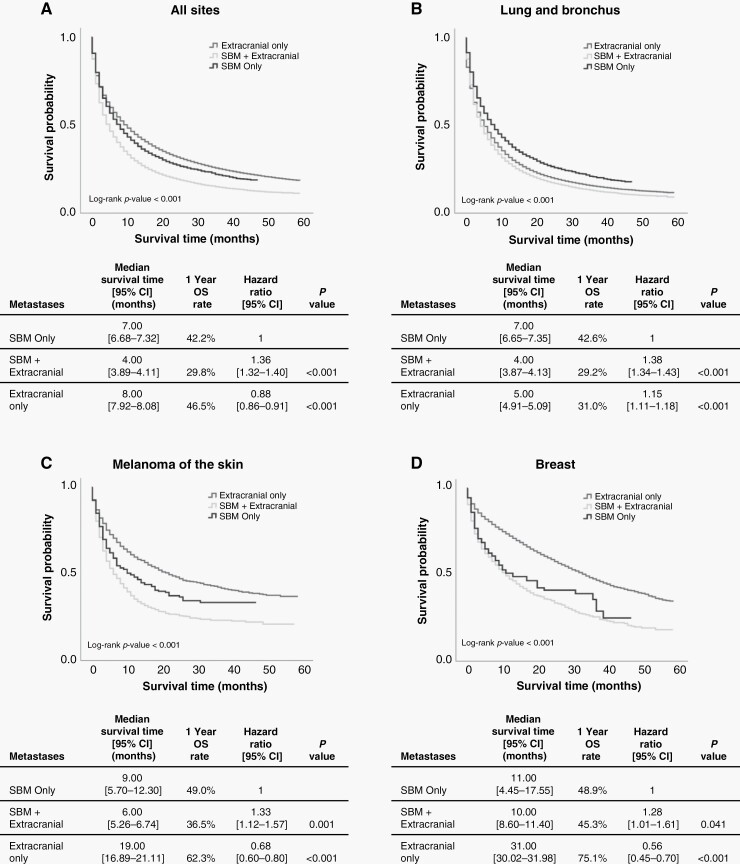
Kaplan-Meier survival curves and survival differences in patients with sBM only, sBM and synchronous extracranial metastases, and synchronous extracranial metastases only in (A) all sites, (B) lung and bronchus, (C) melanoma of the skin, and (D) breast. One-year survival rates are age standardized. Hazard ratios were calculated with multivariate Cox regression, adjusted for sex, race, age, and year of diagnosis.

We evaluated differences in survival outcomes of patients with sBM only, sBM with synchronous extracranial metastases, and synchronous extracranial metastases alone based on the primary tumor site. Kaplan-Meier survival curves comparing patients by the metastases identified at diagnoses for all sites and the 3 primary sites with the highest frequency of sBM are presented in Figure 2. The median survival of patients with sBM for all primary sites was 5.0 [95% CI: 4.9–5.1] and the 1-year OS rate was 29.3%. Using a multivariate Cox regression controlling for potential demographic confounders, we noted significantly poorer survival outcomes in patients with sBM relative to patients with extracranial metastases alone (HR: 1.40 [95% CI: 1.39–1.42], *P* < .001). Interestingly, in patients with primary tumors of the lung and bronchus, having sBM was only associated with an 8.1% increase in the hazard of death relative to having extracranial metastases only (*P* < .001). Patients with lung and bronchus primaries with synchronous extracranial metastases alone had significantly poorer OS than those with sBM only (HR: 1.15 [95% CI: 1.11–1.18], *P* < .001). Patients with small-cell lung tumors and sBM had a nonsignificant 1-year OS advantage compared to patients with non-small-cell lung tumors and sBM (20.9% vs 18.7%). With respect to the 5 primary sites most commonly associated with sBM, breast cancers with sBM had the best median survival time (10.00 [95% CI: 8.6–11.3]) and 1-year survival rate (42.0%). Stratified by subtype, patients with triple-negative breast tumors and sBM had significantly lower 1-year OS rates (24.7%) than patients with HR−/HER2+ (51.5%), HR+/HER2− (55.5%), and HR+/HER2+ tumors (54.2%, *P* < .001). Using a multivariate cox proportional hazards model adjusted for age, sex, race, and year of diagnosis, we found poorer OS in patients with HER2− breast cancers compared to patients with HER2+ breast cancers (HR: 1.15 [95% CI: 1.08–1.22], *P* < .001).

### Survival of Patients With SBM by Demographic and Clinical Characteristics

A multivariate cox proportional hazards regression, adjusted for age, sex, year of diagnosis, annual income, and primary tumor site, was used to determine survival differences between white and nonwhite patients with sBM. We observed a significantly lower hazard of death in white patients relative to nonwhite patients with sBM (HR: 0.91 [95% CI: 0.90–0.94], *P* < .001). Relative to white patients, Black patients had significantly worse OS (HR: 1.04 [95% CI: 1.01–1.09], *P* = .025). American Indian and Alaska Native patients had a higher hazard of death than white patients (HR: 1.15 [95% CI: 1.00–1.32], *P* = .045). Asian and Pacific Islander patients had improved OS relative to white patients (HR: 0.74 [95% CI: 0.71–0.77], *P* < .001). After adjusting for race, year of diagnosis, and the primary site, we noted increased OS in females, compared to males (HR: 0.85 [95% CI: 0.83–0.87], *P* < .001). With regard to age, older adults with sBM had reduced OS relative to adolescents/young adults with sBM (HR:1.50 [95% CI: 1.39–1.59], *P* < .001) and children with sBM (HR: 1.74 [95% CI: 1.51–1.86], *P* < .001). Median survival was 4.0 months [95% CI: 3.88–4.12 in older] in adults with sBM, 15.0 months [95% CI: 12.24–17.76] in adolescents and young adults, and 35.0 months [95% CI: 21.90–48.09] in children. Median household income also exhibited an effect on OS in patients with sBM. Patients with sBM with a median household income less than $75,000 had lower OS than patients with sBM and a median household income greater than $75,000 (HR: 1.16 [95% CI: 1.13–1.19], *P <* .001). Interestingly, the effect of sBM on OS was not constant from 2015 to 2019. Patients diagnosed in 2019 had better OS than patients diagnosed in 2015 (HR: 0.76 [95% CI: 0.70–0.81], *P* < .001). With regard to the impact of additional clinical characteristics on survival outcomes in patients with sBM, the presence of liver, lung, or bone metastases were associated with poorer OS (HR: 1.38, 1.10, 1.05, respectively, *P* < .001).

## Discussion

BM constitute a major source of morbidity and mortality as well as significant pharmacy and hospital expenditures nationwide.^13^ While the true incidence of BM remains unknown, their impact in cancer patients will likely rise with improved systemic therapies, which prolong the survival of patients with primary malignancies, consequently increasing the number of people at risk for developing BM.^14–16^ In this population-based cohort study of the most recently released SEER registry, we present an update on the incidence rate, absolute number, and prognosis of patients with sBM by primary site and by sociodemographic characteristics. Because of the generalizability of the SEER program, which encompasses roughly 27% of the US population, our results have potential applications for screening and diagnostic protocols for sBM in cancer patients.

SEER-based studies that have reported the incidence of sBM are scant. In their seminal work, Cagney et al. found sBM in 2.0% of all patients with malignant cancer and 12.1% of patients with metastatic disease diagnosed between 2010 and 2013.^10^ In the most recently published SEER-based exploration of the epidemiology of sBM, Singh et al. reported an incidence of sBM of 7 per 100,000 persons from 2010 to 2015.^7^ Our study supports these results based on updated SEER data, as we observed sBM in 1.9% of all patients with malignant cancer and an incidence of sBM of 7 per 100,000 persons. Together, our analyses suggest that the incidence of sBM has plateaued from 2010 to 2019. Concurrently, the United States Cancer Statistics Working Group reported the incidence of new cancer diagnoses remained nearly stagnant at 438.6–456.8 per 100,000 persons from 2015 to 2019.^17^ As BM arise from the spread of primary malignancies, decreasing the incidence of sBM requires identifying primaries in early stages via routine cancer screening. This notion is supported by Lefeuvre et al.’s study on breast cancer screening, in which his group found a lower rate of BM in women who underwent screening mammography every 2 years than women who underwent symptomatic mammography.^18^ Similar findings were seen in lung cancer, where those diagnosed via low-dose computed tomography (CT) screening had a nearly 50% lower 3-year BM incidence.^19^ Previous studies have described an underutilization of preventative screening in the past decade for primary neoplasms.^20,21^ Investigators have proposed strategies such as patient navigation programs, phone-call reminders, and monetary incentives to increase cancer screening utilization.^22,23^ Our study suggests that efforts to increase screening should be targeted to patients at risk for lung and bronchus, melanoma, breast, kidney and renal pelvis, and colorectal neoplasms, which were associated with the highest incidence of sBM. Systematic intracranial screening MRI at diagnosis, or even routine screening for patients at-risk of these malignancies may be justified based on our findings. Prospective studies assessing the clinical utility of screening brain MRIs in patients at high risk for breast cancer are ongoing (Dana-Farber Cancer Institute, unpublished data). In addition, further investigations to categorize barriers to routine cancer screening are warranted to develop effective strategies to increase screening.

Previous studies have also found lung and bronchus to be the most common primary cancer site associated with BM. Cagney et al. and Singh et al. reported lung cancers to comprise roughly 80% of sBM, with small-cell lung cancer having a higher prevalence of sBM than other lung cancer subtypes.^7,10^ In the present study, patients with small-cell lung cancer were nearly three times more likely to have sBM than patients with non-small-cell lung cancer. The high prevalence of BM in patients with small-cell lung cancer has been used to support the role of prophylactic cranial irradiation (PCI) and routine intracranial screening in the treatment of small-cell lung cancer, which has been shown to improve quality-adjusted life expectancy.^24–26^ Of note, PCI was established in the “CT era” when asymptomatic sub-centimeter metastases could not be reliably detected and is now being reevaluated in the context of magnetic resonance imaging (MRI) surveillance in an ongoing trial (Southwest Oncology Group, unpublished data). In contrast, in patients with non-small-cell lung cancer, PCI has been shown to significantly reduce the incidence of BM, but with limited benefit to OS.^27^ In our study, OS did not differ significantly in patients with small-cell lung cancer with sBM relative to patients with non-small-cell lung cancer with sBM.

The prognosis of patients with BM remains poor largely due to challenges in delivering systemic therapies across the blood-brain barrier.^28^ We noted that patients with sBM had worse OS than patients with extracranial metastases only. Median survival of patients with sBM in all sites was 5 months. Other contemporary studies utilizing the SEER program have indicated a median survival of 12 months or less across all primary sites, with breast cancer having the highest median survival time.^7,10^ Following these trends, we found that breast cancer patients with sBM had the highest 1-year survival compared to other common primaries with sBM. Interestingly, in a single institutional analysis, Mills et al. found patients with breast cancer to present with a greater number of BM and have poorer OS following BM diagnosis than patients with non-small cell lung cancer and melanoma.^29^ The difference in our results may be explained by the higher proportion of triple-negative breast cancers in Mills et al.’s study. The development of BM in patients with breast cancer varies by subtype, with HER2+ and triple-negative breast cancers having the highest propensity of metastasizing to the brain.^30,31^ In this cohort, patients with HER2+ breast cancer had better survival than patients with HER2− breast cancer. Several other studies have reported a survival advantage in patients with HER2+ breast cancer, which may reflect improved disease control related to targeted therapies for HER2+ tumors.^32,33^ On the other hand the lack of estrogen, progesterone, and human epidermal growth factor 2 receptor expression renders triple-negative breast cancers nonresponsive to targeted therapies, resulting in higher rates of metastases and poorer survival.^34^ Research for new therapies are needed and ongoing. For example, recent preclinical models have demonstrated some efficacy in the use of paclitaxel-loaded polylactide-co-glycolide polyethylene glycol nanoparticles to target triple-negative breast cancer BM.^35^

This paper highlights a population of newly diagnosed cancer patients with worse survival who may benefit from improved treatment strategies. For most patients with sBM, radiotherapeutic or surgical management of BM is prioritized and there may be a meaningful delay in initiating systemic therapy needed for the treatment of primary disease or extracranial metastases. Although the impact of this delay in OS is outside the scope of this paper, increasing utilization of stereotactic radiosurgery, rather than whole brain radiotherapy, may allow more rapid initiation of systemic therapies.^36^ Additional studies are warranted to determine the optimal integration and timing of chemotherapeutic drugs and radiosurgery for patients with sBM. Improved treatment of bulk disease including surgery or novel radio-enhancing drugs may improve outcomes for these patients in the future. Furthermore, improved care coordination between multidisciplinary teams to reduce the time from surgery to adjuvant irradiation of BM and delivery of systemic antineoplastic therapies may elicit better disease control.^37^

We also investigated sociodemographic determinants of survival outcomes in patients with sBM. Nonwhite race and lower socioeconomic status (SES) were independently associated with lower survival. These disparities were also noted in Singh et al.’s study on sBM as well as several other neuro-oncology studies published in the last 2 decades.^38^ Prior literature demonstrates that African Americans are more likely to have sBM and experience in-hospital mortality following brain tumor resection and less likely to receive stereotactic radiosurgery or participate in clinical trials than white patients. Epidemiological studies have also reported lower utilization of screening, less cancer symptom awareness, and cancer-help-seeking behaviors in patients with lower SES.^39,40^ Moreover, patients with BM with lower SES receive radiation and chemotherapy less frequently.^41^ We also observed poorer OS in males and older adults. Previous studies have suggested that elderly patients derive less benefit from aggressive intracranial treatments than their young adult counterparts.^42^ Finally, we noted improved OS in patients diagnosed in 2019 than those diagnosed in 2015. In their analysis of SEER data, Singh et al. also noted improved OS in patients diagnosed more recently.^7^ This trajectory may reflect improvements in therapy for patients with metastatic disease that occurred after 2015, such as the establishment of immunotherapy for patients with lung cancer and melanoma.^43^ Future efforts aimed at increasing access to these therapies in older, lower SES, and nonwhite populations are imperative to mitigate the disparities observed in this study.

We recognize important limitations to this study. First, the lack of data in SEER capturing the development of BM after diagnosis prevents us from reporting the true incidence of BM. Furthermore, the utilization of intracranial screening imaging varies by the primary tumor site. The National Comprehensive Cancer Network (NCCN) recommends screening for asympomatic BM at the time of initial diagnosis for lung cancer and melanoma.^44,45^ Thus, there may be a relative overestimation of synchronous BM in these primary cancers. On the other hand, the NCCN recommends screening MRIs only for patients with recurrent or stage IV breast cancer who present with neurologic symptoms, which may result in an underestimation of sBM in this population.^46^ Next, the resolution of the imaging modality used to screen for BM can impact the likelihood of identifying BM, with fluorodeoxyglucose positron emission tomography and 3D MRI sequences likely being superior to 2D, and MRI being superior to CT.^47^ SEER does not collect information on the imaging modality used to screen for sBM, therefore we could not control for the screening imaging method used in our analysis. Additional limitations inherent to SEER-based studies include the lack of data on the number and size of BM, brain-directed treatment, systemic therapies, type of healthcare center, or tumor recurrences following initial diagnosis, which can have a significant impact on survival outcomes. Thus, our survival analysis is limited by this lack of data. The exclusion of patients with 0 days of follow-up may also introduce selection bias to our analysis, as patients with 0 days of follow-up may have been too ill to receive care. However, this bias is likely minimal because NCI requires at least 95% complete follow-up from participating registries.^48^ Finally, while our study has high external validity due to the utilization of a population-wide registry, the generalizability is limited by minor differences in the population represented in SEER and the US population. According to NCI, there is a slight overrepresentation of foreign-born immigrants and individuals with less than a high school diploma in SEER. Additionally, our incidence rates were adjusted to the 2000 US Population, in accordance with the SEER*Stat software; however, this limits the accuracy of the incidence relative to the current US population.

Despite these limitations, our study provides the most updated trends in sBM in the United States, which has implications for patients with malignant disease, providers, and healthcare systems. Building multi-institutional databases to enable future studies of the development of metachronous BM are merited to better comprehend the impact of BM in the disease progression and survival of patients with cancer.

## Conclusion

The incidence rate of sBM in 2015–2019 has remained stable, and most cases continue to be associated with primary lung and bronchus cancers. The presence of sBM is associated with poor prognoses. The median survival time of all sBM patients was 5 months, which has remained stable. Among patients with sBM, white, female, young, or higher household income patients and those with breast cancer primaries have better OS. Continuing studies characterizing the epidemiology of BM are necessary to inform the improvement of screening, diagnostic, and treatment standards.

## Supplementary Material

vdad015_suppl_Supplementary_Tables
