# Supplementary material for: Epidemiological trends, prognostic factors, and survival outcomes of synchronous brain metastases from 2015 to 2019: a population-based study
Source: Neurooncol Adv. 2023 Mar 5;5(1):vdad015. doi: 10.1093/noajnl/vdad015 (PMC10034914; doi:10.1093/noajnl/vdad015)
Supplement: vdad015_suppl_Supplementary_Tables [file vdad015_suppl_supplementary_tables.docx]

| Supplementary Table 1. Characteristics of Patients with SBM Only Compared to Patients with Both SBM and Extracranial Metastases | | |  |
| --- | --- | --- | --- |
|  | **Brain Metastases Only**  **(N = 8484)** | **Brain Metastases and Extracranial Metastases**  **(N = 23889)** | ***P*-value** |
| Age, Mean ± SD | 64.8 ± 11.5 | 63.2 ± 12.0 | <0.001 |
| Age Category |  |  |  |
| Children | 29 (0.3%) | 63 (0.3%) | 0.264 |
| Adolescents and Young Adults | 104 (1.2%) | 575 (2.4%) | <0.001 |
| Older Adults | 8351 (98.4%) | 23251 (97.3%) | <0.001 |
| Sex |  |  |  |
| Females | 4097 (48.2%) | 11463 (48.0%) | 0.627 |
| Males | 4387 (51.7%) | 12426 (52.0%) | 0.627 |
| Race |  |  |  |
| White | 6687 (78.8%) | 18790 (78.7%) | 0.752 |
| Black | 1060 (12.5%) | 2540 (10.6%) | <0.001 |
| Asian or Pacific Islander | 56 (0.7%) | 2290 (9.6%) | <0.001 |
| American Indian/Alaska Native | 646 (7.6%) | 182 (0.8%) | <0.001 |
| Year of Diagnosis |  |  |  |
| 2015^1^ | - | 3895 (16.2%) |  |
| 2016 | 2009 (23.7%) | 4830 (20.2%) | <0.001 |
| 2017 | 2263 (26.7%) | 5121 (21.4%) | <0.001 |
| 2018 | 2077 (24.5%) | 5003 (20.9%) | <0.001 |
| 2019 | 2135 (25.1%) | 5076 (21.2%) | <0.001 |
| Primary Site |  |  |  |
| Lung and Bronchus | 7067 (83.3%) | 17579 (73.6%) | <0.001 |
| Small-cell | 1017 (12.0%) | 2903 (12.2%) | 0.689 |
| Non-small-cell | 377 (4.4%) | 918 (3.8%) | 0.015 |
| Melanoma | 278 (3.3%) | 1074 (4.5%) | <0.001 |
| Breast | 137 (1.6%) | 1167 (4.9%) | <0.001 |
| HR-/HER2- | 29 (0.3%) | 194 (0.8%) | <0.001 |
| HR-/HER2+ | 14 (0.2%) | 138 (0.6%) | <0.001 |
| HR+/HER2- | 48 (0.6%) | 468 (2.0%) | <0.001 |
| HR+/HER2+ | 15 (0.2%) | 189 (0.8%) | <0.001 |
| Kidney and Renal Pelvis | 126 (1.5%) | 914 (3.8%) | <0.001 |
| Colorectal | 70 (0.8%) | 427 (1.8%) | <0.001 |
| Esophagus | 84 (0.9%) | 264 (1.1%) | 0.378 |
| Pancreas | 33 (0.4%) | 228 (1.0%) | <0.001 |
| Prostate | 20 (0.2%) | 213 (0.9%) | <0.001 |
| Stomach | 27 (0.3%) | 161 (0.7%) | <0.001 |
| Liver | 27 (0.3%) | 99 (0.4%) | 0.222 |
| Urinary Bladder | 23 (0.3%) | 85 (0.4%) | 0.245 |
| Testis | 5 (0.01%) | 107 (0.4%) | <0.001 |
| Thyroid | 10 (0.1%) | 76 (0.3%) | 0.002 |
| Cervix | 11 (0.1%) | 50 (0.2%) | 0.146 |
| Ovary | 11 (0.1%) | 54 (0.2%) | 0.088 |
| ^1^The presence of distant lymph node metastases and other metastases was not recorded in the SEER database for patients diagnosed in 2015. Therefore, the number of patients with sBM only could not be calculated for patients diagnosed in 2015. | | | |

| Supplementary Table 2. Incidence^1^ of SBM by Primary Site from 2015-2019 Stratified by Age Category | | | |
| --- | --- | --- | --- |
| Primary Tumor Site | **Children**  **(0-14 years)** | **Adolescents and Young Adults**  **(15-39 years)** | **Older Adults**  **(40+ years)** |
| All Sites | 0.144 | 0.510 | 16.034 |
| Lung and Bronchus | 0.002 | 0.180 | 12.401 |
| Melanoma | 0.001 | 0.054 | 0.676 |
| Breast | 0.000 | 0.065 | 0.597 |
| Kidney and Renal Pelvis | 0.005 | 0.014 | 0.489 |
| Colorectal | 0.000 | 0.011 | 0.239 |
| Esophagus | 0.000 | 0.004 | 0.168 |
| Pancreas | 0.000 | 0.000 | 0.124 |
| Prostate | 0.000 | 0.000 | 0.110 |
| Stomach | 0.000 | 0.010 | 0.089 |
| Liver | 0.001 | 0.001 | 0.059 |
| Urinary Bladder | 0.000 | 0.002 | 0.053 |
| Ovary | 0.000 | 0.005 | 0.031 |
| Thyroid | 0.000 | 0.006 | 0.042 |
| Cervix | 0.000 | 0.007 | 0.027 |
| ^1^All incidence rates are per 100,000 and age-adjusted to the 2000 US standard population | | | |
